# The role of photobiomodulation when associated with microneedling in female pattern hair loss: A randomized, double blind, parallel group, three arm, clinical study protocol

**DOI:** 10.1097/MD.0000000000014938

**Published:** 2019-03-22

**Authors:** Suzana Polonca da Silveira, Sandra Rojas Urquizas Moita, Silvia Vicente da Silva, Maria Fernanda Setúbal Destro Rodrigues, Daniela de Fátima Teixeira da Silva, Christiane Pavani

**Affiliations:** a Biophotonics Applied to Health Sciences Postgraduation Program, Universidade Nove de Julho, Universidade Nove de Julho; b Duetto Hair; c Escola de Saúde e Administração - Essa, São Paulo, Brazil.

**Keywords:** androgenetic alopecia, female pattern hair loss, microneedling, photobiomodulation, telogen effluvium

## Abstract

**Abstract:**

The female hair loss pattern was originally described as a synonym for androgenetic alopecia. It has been defined as progressive miniaturizations of the hair follicles, with a great impact on the quality of life of affected patients, causing significant psychosocial limitations. It was recently proven that photobiomodulation is a safe and effective way to treat the different types of hair loss. It was also known that microneedling is a minimally invasive dermatological procedure that is applied to a wide range of dermatological conditions, including androgenic alopecia, telogen effluvium, as well as other facial and bodily conditions.

**Goal:**

The aim of this study is to verify if there is an increase in the capillary density of strands of hair, when combining 660 nm red laser photobiomodulation and microneedling in addressing female pattern hair loss (FPHL).

**Methods:**

There will be 66 patients divided into 3 treatment groups. G1: microneedling and 660 nm red laser photobiomodulation *sham*; G2: 660 nm red laser photobiomodulation and microneedling *sham*; G3: microneedling and 660 nm red laser photobiomodulation. The treatment will consist in 36 sessions, 3 times a week for 3 consecutive months, with an insertion of microneedling every 30 days. The patients and the researchers will be blinded. The patients will be evaluated before, during, and after the treatments, by digital photography and the trichoscopy method (dermoscopic imaging of the scalp and hair).

**Expected Results:**

It is expected that differences will be found in the growth rates of a strand of hair in mm/d, in the density of a strand of hair in n/cm^2^, in the diameter of a strand of hair, as well as in the anagen/telogen ratio.

**Ethics and dissemination:**

This protocol was approved by the Research Ethics Committee of the Nove de Julho University, São Paulo, Brazil, on the date of November 28, 2018 (CAAE: 01381718.0.0000.5511 - Acceptance Number: 3044061). This trial has been registered with the Brazilian Registry of Clinical Trials (REBEC TRIAL RBR-76VCCV). This study is not yet recruiting. Issue date: February 20, 2019.

## 1 Introduction

Female pattern hair loss (FPHL) has emerged as the preferred term for androgenetic alopecia (AGA) in women, due to the uncertain relationship between androgens and this condition.^[1,2]^ FPHL is a common cause of alopecia in women. It affects 29% to 38% of women, as well as >55% of women over 70 years. However, it can start at any age after puberty. The FPHL prevalence in women aged under 50 years ranges from 6% to 12% and this increases after the menopause, suggesting a possible hormonal influence. In the United States alone, FPHL affects over 21 million women.^[3]^

Each strand of hair is made up of a hair follicle, being a mini complex skin organ, which is named the pilosebaceous unit, in addition to its associated structures, such as a sebaceous gland, an apocrine sweat gland, and an erector muscle of the hair.^[4]^ Hair growth occurs periodically in a cycle consisting of 3 distinct phases: anagen, catagen, and telogen. The length of each phase is regulated by intrinsic and extrinsic factors throughout life and it is influenced by physiological and pathological conditions.^[5,6]^ In a female pattern hair loss, there are alterations in the dynamics of the capillary cycle, whereby the anagen phase shortens and the telogen phase lengthens. Thus, in each cycle, there is a miniaturization of the hair. In addition to length, the diameter of a strand of hair is also compromised and finally, at the end of this procedure, there is a gradual replacement of long hair by short and thin hair. As a result, alternative strategies are necessary and they are urgently required for the treatment of hair loss.^[7]^

It is known today that photobiomodulation (both with a laser and with light emitting diode—LED) is used to treat in a non-destructive and non-thermal manner, a variety of biological targets that participate in the regulation and in the differentiation of the germ cells, producing stimuli for the proliferation and the differentiation of the follicular stem cells (the bulge cells), into these germ cells. In other words, laser and LED photobiomodulation make the difference in hair growth, by stimulating the capillary germ cells.^[8,9]^

Photobiomodulation is a technology that is used to treat a plethora of conditions, which require a stimulation for wound healing, for relieving pain, for inflammation, and for the restoration of many other functions. The skin is an organ that is naturally exposed to light more than any other organ. It responds to the red wavelengths and the near infrared. Most of the cell chromophores, including the flavins, iron and sulfur, are localized in the mitochondria.^[8,9]^ Photons are absorbed by cytochrome C oxidase, a chromophore that is present in the mitochondria of the cells in the skin. Consequently, the transport of electrons, a release of nitric oxide and adenosine triphosphate, an increased blood flow and an increase of the reactive oxygen species, all occur. Thus, the stem cells can be activated, allowing for a greater repair and a healing of the tissues.^[5]^ Photobiomodulation induces a photochemical reaction in the cells, without producing any thermal effect, or causing any irreversible damage. Effects, such as a proliferation, a migration, an oxygenation, and an adhesion, are all induced and this may allow for the growth of the hair, by promoting the exit of the follicle from the telogen phase and stimulating the active anagen phase.^[6]^ Photobiomodulation seems to be a promising noninvasive treatment for adults with alopecia, which is safe for both the office and home care. At this time, photobiomodulation has proven itself to be an attractive alternative for the treatment of alopecia, especially for those individuals who do not wish to proceed with pharmaceuticals or with surgical treatments.^[10]^

Microneedling, which is also known as percutaneous collagen induction therapy, is a minimally invasive procedure that creates micro-holes in the skin, increasing the permeability of drugs and actives, stimulating the production of collagen, while at the same time, it improves noncicatricial alopecia, without causing the total de-epithelialization that is observed in the ablative techniques.^[9,11,12]^ The technique acts in 2 main ways: a percutaneous induction of collagen through the response of the inflammatory process, while at the same time, preserving the epidermis and promoting the normal formation of collagen and elastin in the dermis. The percutaneous induction of collagen brings us closer to the ideal^[13–15]^ and it facilitates the Transdermal Ingredient Access System known as “Drug Delivery,” since it breaks down the properties of the epidermal barrier, which is the cause of difficulties for the transdermal delivery of therapeutical agents.^[13,16–18]^ Through the micropunctures, lesions will be triggered, which will promote an increase of the platelet-derived growth factors, the transforming alpha and beta growth factors (TGFα, TGFβ), the vascular endothelial growth factor (VEGF), the fibroblast growth factor (FGF), the epidermal growth factor (EGF), the Wnt proteins, among others, without significantly damaging the epidermis. It is known that the Wnt/β-catenin signaling pathway mediates the hair morphogenesis and the capillary stem growth, making the technique effective for this condition.^[9,12,13,19]^

There is currently a trend towards an indication of less invasive procedures, which are isolated or in an association, aiming at reducing the risk of complications, with earlier return to work activities. Microneedling becomes a promising therapeutical resource and it can be used in conjunction with cosmetics, while assisting in the penetration of assets.^[20]^ In the light of all of this information, the objective of this double-blind randomized clinical study will be to verify if there is an increase in hair density (the number of strands of hair per cm^2^), together with the association of photobiomodulation with red laser and microneedling, in the improvement of female pattern hair loss—FPHL.

## 2 Method

### 2.1 Study design

This will be a clinical, randomized, comparative, double-blind study. It will be implemented over 12 weeks, divided into 36 sessions, 3 times a week. It will be conducted at the Biophotonics Outpatient Clinic of the Nove de Julho University, in the city of São Paulo, Brazil. This protocol was written and was based on Standard Protocol Items: Recommendations for Interventional Trials (SPIRIT). The dissemination and the registration for participation in the study will be carried out through the Nove de Julho University website. The participants will be residents who will mainly be recruited in the city of São Paulo. The participants will be informed about the research, the procedures, the risks, and the benefits by SPS (author of this protocol). If they agree, they will sign the informed consent form (ICF). Only the participants, who after reading and agreeing to the protocol and who sign the ICF, will be part of the study, following the schedule as described in Table 1.

**Table 1:**
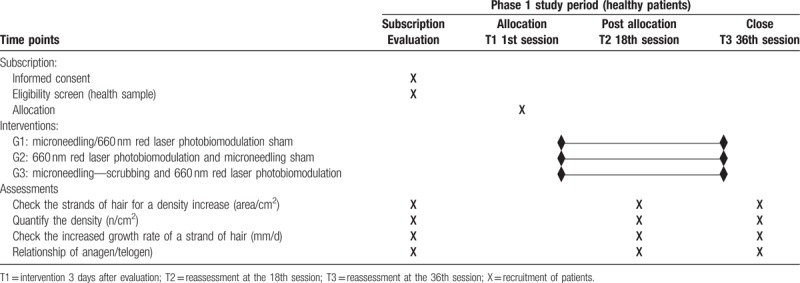
Registration schedule, interventions, and treatment evaluations.

The study will last for 2 years, starting from March 2019. The study is not yet recruiting. After the recruitment, the researchers will check if the patient meets the inclusion/exclusion criteria based upon the anamnesis questionnaire and the capillary evaluation. With regard to personal and social daily habits, the anamnesis questionnaire will include information about food, water intake, smoking, sun exposure, sleep quality, the treatments as applied by professional hairdressers, such as hair dye, or the procedures that include use of hair chemistry, the use homecare products, and so forth. The investigators will check if the patient meets the inclusion/exclusion criteria based upon the anamnesis questionnaire, together with an evaluation of their scalp.

The patients and/or the public will not be involved in the design, the recruitment or the conduct of the study. The participants will be women with a capillary loss, or a fall of hair and who suffer from female androgenetic alopecia and telogen effluvium, as is described in FPHL.

### 2.2 Patient and public involvement statement

The patients and/or the public will not be involved in the design, the recruitment, or the conduct of the study. The participants will be women with a capillary loss, or a fall of hair and who suffer from female androgenetic alopecia and telogen effluvium.

### 2.3 Inclusion criteria

This study will be conducted on women aged 18 to 60 years: who suffer from a female pattern hair loss, together with a telogen effluvium and an androgenetic alopecia profile; on women with a capillary rarefaction; on women with a great hair loss during the last 5 years; and on women with a gradual hair loss in the last year. Finally, they must not have performed any chemical procedures on their hair during a period of the last 3 months.

### 2.4 Exclusion criteria

Participants will be excluded from the survey if they present any of the following:

- scarring diseases in their scalp,
- wear wigs or appliqués that cause a weight on the stem,
- use drugs continuously as anti-inflammatories, anxiolytics, antifungals, hormone inhibitors,
- have fungal or inflammatory conditions,
- women who participate in a hormonal gynecological treatment,
- have uncontrolled chronic diseases like Systolic Arterial Hypertension and diabetes (increased blood sugar),
- participants are unable to understand and sign the ICF.

### 2.5 Sample size calculation

The sample size was calculated based on a study by Kim in 2013.^[21]^ This was a randomized, double-blind, placebo-controlled, 24-week, 40 male and female subject study, who were suffering with AGA and who were recruited and programmed to receive a treatment by using a domesticated photobiomodulation when using a helmet device, with wavelengths of 630, 650, and 660 nm, together with a simulated device for 18 minutes per day. The study evaluated the patients by using a phototrichogram, with an assessment of the density, the hair thickness, and an evaluation of the global growth rate of the hair. The highest and the lowest values of hair density percentages in cm^2^ (amount of hair present in a square centimeter) were obtained, as well as for the standard deviation of the measurements. The worst scenarios were used for their calculation. The lowest and the highest values were 17.2 and −2.1, respectively; the largest standard deviation was 18.3 and the number of treatment groups was 2. These values were used to calculate the effect size as follows: 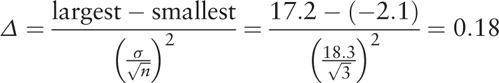


By using the effect size value as calculated above, the sample size was calculated by G∗ Power Software (V.3.1.9.2, Dusseldorf, Germany) using *F* test (analysis of variance [ANOVA] repeated measurements, within-between interactions), test power of 80%, single-tailed test was 5%, 3 study groups, and 3 measurements resulted in 66 patients.

### 2.6 Randomization

The randomization will be performed by a researcher (MFSDR, author of this protocol) who will not be directly involved in the treatments of the participants. The same randomization will be generated in the Excel 2013 Program (Microsoft, Washington). Opaque envelopes will be identified with sequential numbers and they will each receive a paper containing the information of which treatment will be performed in the specific patient, according to the draw. These envelopes will be sealed, stored safely, and held confidentially, by the same researcher who generated the randomization. Immediately prior to the treatments, the investigator responsible for each treatment will receive an envelope in sequence and then perform the indicated procedure.

### 2.7 Intervention

In order to carry out this study, a target thinning area of 2 cm in diameter at the temporal region of the scalp will be scraped. Subsequently, analyzes will be obtained through the evaluation tools that encompass various procedures, such as global photography and trichoscopy. For the evaluations to be performed, these patients must be present 3 days prior to the start of scaling, labeling, and evaluation. After 3 days, the treatments will commence as described above. Following the indication of randomization, the participants will receive a procedure as proposed for their group: G1—microneedling with 0.5 mm needles and a 660 nm red laser photobiomodulation sham (a beep will be triggered for the laser simulation); G2—660 nm red laser photobiomodulation and a microneedling sham (pen at 0.0 mm, without touching the skin, just simulating the movements in the intervention); and G3—microneedling with 0.5 mm needles + 660 nm red laser photobiomodulation (i.e., everything will work, there will be no sham treatments involved, allowing the blindness of the participant).

For the interventions, the participants will have their scalp anesthetized with lidocaine 5% on their frontal and temporal regions, without any previous hygiene. This anesthetic will be in contact with the skin for 20 minutes. It will be removed after this period by a solution of 2% chlorhexidine, so that a local antisepsis occurs. After the total anesthetic removal and the antisepsis of this region, the participants will be submitted to a technique as proposed for each group and as described above. An electronic pen, the Derma Erase Pen Model DE77 of the brand VR Medical will be introduced, with tips composed of 42 needles, with the pen employing a speed adjustment and with sizes of 0.0 to 2.0 mm needles. The choice for this project will be with an adjustment of 0.5 mm in length. The equipment will be passed in longitudinal, vertical, and diagonal directions, until a slight erythema, preceded by a petechiae, and a bleeding dew is observed, which will be considered to be the end point of the procedure. After the procedures, the regions will be sanitized with a sterile gauze soaked in filtered water. Following, the participants will also receive 660 nm red laser photobiomodulation on their scalps, at points within a range of 1.5 cm away.^[22,23]^ The number of irradiated points will vary according to size of the temporo-parietal region affected by the hair loss. The equipment used will be the Elite Olympus Laser (DMC), with a 660 nm wavelength, including 100 mW power, 5.6 W/cm^2^ irradiance, 40 seconds exposure time per point, 4 J radiant energy per point (226 J/cm^2^).

**Figure 1:**
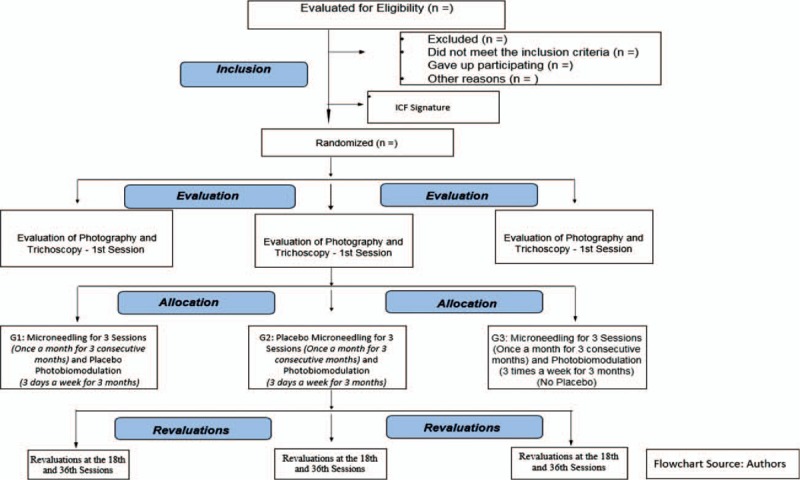
Flowchart of the study.

During the ensuing routines, the patients will undergo 3 months of treatment and they will receive 36 sessions of photobiomodulation (3 times a week) and 3 sessions of microneedling (1 session per month)—in other words, with an interval of 30 days between them. It is important to note that for G1 and G2, there will be sham applications for the photobiomodulation and the microneedling, respectively. SPS, author of this study protocol, will perform these treatments. The flowchart of this study, with treatment groups and evaluations are presented in Fig. 1. During the sessions the researcher (SPS) will remind the participants the importance of adherence to the treatment sessions in order to improve adherence to the intervention protocol. The participants will be instructed to continue taking their medications for health conditions as usual.

### 2.8 Study variables

The primary variable of this study will be the density of the strands of hair per area in cm^2^. The secondary variables will be the growth rate of a strand of hair (mm/d), the hair's diameter (μm), the ratio of anagen/telogen and the quality of life of the participants. Global scalp photographs will also be recorded for a capillary evaluation, according to the classification of Ludwig (3-point scale) and Sinclair (5-point scale).

### 2.9 Global photos

The overall photos will be used to observe the hair thinning in the affected scalp and the evaluator (SRUM, author of this work) will follow the standard of Ludwig for comparison purposes. Despite being a subjective method, this will provide for a standardization of the hair evaluations in practice and in this particular clinical trial, involving the participants with FPHL. The overall pictures/images will be performed by positioning a device, whereupon the chin and the patient's forehead will be fixed, thus, ensuring that the pictures are always held in the same position and in the same parameter. The camera and the lighting system will be by professional devices that guarantee an image quality (Canon 6D Mark II Flash Canon Camera, 24 × 70 mm Canon Lens), which will be fixed and standardized by professional photographer settings, in order to always ensure that the same illumination and parameters are used in each of the days of assessments. These photos will be used later by the blinded evaluator (SRUM, author of this protocol), who will compare a series of photographs of each of these participants, without knowing what methods were employed in the participants.

### 2.10 Trichoscopy

Trichoscopy is the usual term used to describe a videodermatoscopy of the hair and the scalp. It is used to display the hair and the scalp at varying different enlargements, thereby providing pictures that are relevant for a clinical evaluation of a patient. The device is also equipped with software, in order to measure the relevant parameters of the trichology. It will be used to perform a density measurement of the hairs (cm^2^), in order to determine the rate of hair growth (mm/d). It will also be used to determine the anagen/telogen ratio (the ratio of the different phases of the hair) and to measure the hair diameters (μm). This trichoscopic procedure will be carried out by using AM7515MZT Dino-Lite Edge Equipment, managed by MIBEX Trade EIRELI–EPP. It has a polarization feature which allows one to filter the light from LEDs, thereby reducing the reflection from the skin's surface. This will provide for a better visualization of the area to be examined. The trichoscopic equipment will have Dino Capture software bundled with it, in order to assist in the assessments, as well as with ImageJ^©^ software (U. S. National Institutes of Health, Maryland). All of the measurements will be performed by SRUM, who was previously trained by industry representatives and the VR Medical MIPEX Company in Brazil. All of the equipment to be used is appropriate for the legal regulations of Brazil and the equipment will be standardized in the categories of lighting and positioning on the scalp, in order to minimize any experimental bias.

### 2.11 Quality of life

In terms of their quality of life, a health-related Quality of Life questionnaire will be used for those women with androgenetic alopecia (WAA-QOL), with agreement of the owner Merck & Co., Inc.^[24]^ This questionnaire, was translated and submitted to cross-cultural adaption to Brasilian Portuguese.^[25]^ All of the participants will respond to this self-assessment Quality of Life questionnaire, composed of 16 questions. The questionnaires will take a full 10 minutes to be completed. The questionnaires will be applied by SPS. The data that will be collected from this study will only be administered by the principal investigators (the authors of this document).

### 2.12 Statistical analysis and data analysis plan

All the personal records of the participants, such as signed ICF will be stored in locked file cabinets. The information will be transferred to a computer by SPS (author of this protocol) and the participants will be identified by ID numbers. All the collected data will be stored on a university computer, protected by a password and accessed by DdFTdS (author of this protocol) for statistical analysis or the other researchers’ supervisioning this study. The Shapiro-Wilk test will be used to test the normality of the data. If the data are non-parametric, the normalization will be performed by a mathematical strategy. The ANOVA test for the independent variables followed by Bonferroni post-test will be used for the inferential analysis. A *P* < .05 value will be considered statistically significant. DdFTdS will perform all of the statistical analyses.

### 2.13 Expected results

The researchers hope to identify whether there will be an increase in the strand of a hair's density (the number of strands per area in cm^2^) when combining a 660 nm red laser photobiomodulation with microneedling in a FPHL, as assessed by the Trichology Dino-Lite Equipment and the overall image/photo measurements. These measurements will be performed prior to, during and after the 36 treatment sessions. At the primary endpoint and at the secondary endpoints, it is expected to verify improvements in the quantifications of hair strand density (n/cm^2^), as well as to verify increases in the growth rate capillary of the hair strands (mm/d). Finally, it is expected to verify improvements in the anagen/telogen ratio.

## 3 Discussion

This current work is presently describing a study protocol for a randomized, double-blind, clinical trial, in order to study and evaluate the density of strands of hair by area (cm^2^), based upon a comparison of 2 interventions, from 3 different groups. These being: a photobiomodulation and a microneedling sham; a sham photobiomodulation and microneedling; and photobiomodulation and microneedling. The results of this study are expected to provide evidence on the role of phototherapy and microneedling in conditions of a female pattern hair loss.

In this protocol, the choice of the photobiomodulation parameter was made from studies that have demonstrated that an application of red light on the scalp could trigger a proliferation of cells. This would be with an increase of the collagen fibers and a decrement of the metalloproteinases, as well as with an increase in the circulation, modulating the processes of the inflammatory conditions. This would include a growth of new hair and the subsequent reversal of the hair follicles, from the dormant telogen growth stage, into the active growth stage or anagen.^[26–29]^

Similarly, the parameter choice of microneedling occurred on account of scientific papers showing the proposed technique as a mechanism for hair growth in non-scarring alopecia. The technique provides for an outbreak of increased platelet derived growth factors; TGF-α and TGF-β1; VEGF; the fibroblast growth factor (FGF); EGF; the Wnt proteins and so forth, without significantly damaging the epidermis.^[10,13,21,30]^

Studies involving the quality of life have been reported in the scientific literature. For this work, the “QOL choice” came from studies that have presented relevant questions for the quality of life in those women with androgenetic alopecia. The items for this validated questionnaire were generated through a literature review, together with a discussion with clinical specialists, as well as with a focus group that included women with AGA.^[24]^

The inclusion and the exclusion criteria for this study have been based on the literature that objectively formulates what needs to be chosen as the basis for a good study, such as the use of medications, or inadequate chemotherapy, or the patient's ease of following the necessary procedures, in order to achieve an effective result of the treatment applied.^[20,21]^

The strength of this study will be the presence of the 3 combined intervention groups being their own control, since the interventions will be evaluated at the beginning, the middle, and at the end of the study.

The limitations of this study may be related to the life habits and the routines of the participants. These processes can affect the results, as a consequence of their daily stress, their food, their cosmetic use, and their photo exposures.

To the best of the researchers’ knowledge, no previous randomized control trial has evaluated whether phototherapy and microneedling, when associated in the same therapy, can modulate a greater response, or better, when applied together. The current proposed trial is the first randomized clinical trial study to evaluate the role of phototherapy when associated with microneedling in a female pattern hair loss condition. In fact, the results of this study will provide valuable clinical evidence for an objective assessment of the potential benefits and risks of the stated procedures. The final results will be published at the end of the study.

### 3.1 Other information—ethics and disclosure

This protocol was approved by the Research Ethics Committee of the Nove de Julho University, São Paulo, Brazil, dated November 28, 2018 (CAAE: 01381718.0.0000.5511)—Acceptance Number: (3044061), including the Informed Consent Form. The trial has already been registered at the Brazilian Registry of Clinical Trials (REBEC - RBR-76VCCV) first registered at October 11, 2018, UTN: U1111–1222–2308, which provides public access to the full protocol. All the items from the World Health Organization Trial Registration Data Ser are available on the REBEC register website. Any modification regarding the study protocol will be sent to the Research Ethics Committee of the Nove de Julho University as an amendment, and an updated version will be sent to REBEC. After the publication of the protocol, the data will be collected and the results will be presented at meetings and published in a scientific journal, selected by an area of interest and upon the impact factor. At the end of the study, the main results will be 2inated to the participants by email. The authorship of the results manuscript will include the authors of the protocol, together with others who may contribute to the procedures or the data analysis.

## Acknowledgments

The authors would like to thank the VR Medical Company which kindly provided the Electronic Derma Erase Pen; MIPEX for their kind supply of the Dino-Lite Trichology Equipment; and DMC by their kind provision of the Olympus Laser Equipment. Without the support of these companies and for the use of all of this equipment, inevitably, the formulation of this protocol would not even be possible. They also acknowledge Merck & Co., Inc. for the authorization to use the WAA-QoL-BP questionnaire.

## Author contributions

Suzana Polonca da Silveira wrote the original draft, designed the protocol, and will perform the treatments. Sandra Rojas Urquizas Moita will perform que data collection using the equipments and apply the QOL questionnaire to the participants. Suzana Polonca da Silveira critically reviewed and edited the manuscript. Daniela de Fátima Teixeira Silva performed the calculations of the sample size, proposed the statistics and will analyze the data. Maria Fernanda Setúbal Destro Rodrigues generated the randomization and designed the study. Christiane Pavani conceived and designed the treatment protocol, reviewed and edited the manuscript. Christiane Pavani supervises this research. All of the authors have read and approved the final version of this protocol and the manuscript.

**Conceptualization:** Suzana Polonca da Silveira, Maria Fernanda Setúbal Destro Rodrigues, Christiane Pavani, Daniela de Fátima Teixeira da Silva.

**Data curation:** Daniela de Fátima Teixeira Silva.

**Formal analysis:** Daniela de Fátima Teixeira Silva.

**Investigation:** Suzana Polonca da Silveira.

**Methodology:** Sandra Rojas Urquizas Moita, Suzana Polonca da Silveira.

**Project administration:** Christiane Pavani.

**Supervision:** Christiane Pavani.

**Writing – original draft:** Suzana Polonca da Silveira.

**Writing – review & editing:** Silvia Vicente Silva, Christiane Pavani.

Christiane Pavani orcid: 0000-0001-8275-7370.
